# Objective Test of Cochlear Dead Region: Electrophysiologic Approach using Acoustic Change Complex

**DOI:** 10.1038/s41598-018-21754-7

**Published:** 2018-02-26

**Authors:** Soojin Kang, Jihwan Woo, Heesung Park, Carolyn J. Brown, Sung Hwa Hong, Il Joon Moon

**Affiliations:** 1 Department of Otorhinolaryngology-Head and Neck Surgery, Samsung Medical Center, Sungkyunkwan University School of Medicine, Seoul, Korea; 2 School of Electrical Engineering, Biomedical Engineering, University of Ulsan, Ulsan, Korea; 3 Departments of Speech Pathology and Audiology, University of Iowa, Iowa City, Iowa USA; 4 Department of Otorhinolaryngology–Head and Neck Surgery, Samsung Changwon Hospital, Sungkyunkwan University School of Medicine, Changwon, Korea

## Abstract

The goal of this study was to develop an objective and neurophysiologic method of identifying the presence of cochlear dead region (CDR) by combining acoustic change complex (ACC) responses with threshold-equalizing noise (TEN) test. The goal of the first study was to confirm whether ACC could be evoked with TEN stimuli and to also optimize the test conditions. The goal of the second study was to determine whether the TEN-ACC test is capable of detecting CDR(s). The ACC responses were successfully recorded from all study participants. Both behaviorally and electrophysiologically obtained masked thresholds (TEN threshold and TEN-ACC threshold) were similar and below 10 and 12 dB SNR in NH listeners, respectively. HI listeners were divided into HI (non-CDR) and CDR groups based on the behavioral TEN test. For the non-CDR group, TEN-ACC thresholds were below 12 dB which were similar to NH listeners. However, for the CDR group, TEN-ACC thresholds were significantly higher (≥12 dB SNR) than those in the NH and HI groups, indicating that CDR(s) can be objectively detected using the ACC. Results of this study demonstrate that it is possible to detect the presence of CDR using an electrophysiologic method.

## Introduction

Sensorineural hearing loss (SNHL) is often caused by damage to the cochlear hair cells. If hearing loss is relatively mild, the damage may be limited to the outer hair cells. However, if the extent of the hearing loss exceeds approximately 55 dB HL, both outer and inner hair cells will be affected. The inner hair cell loss or dysfunction may create^1–3^ regions of extensive damage on the basilar membrane that are referred to in the literature as “cochlear dead regions” (CDR). Little or no information from the basilar membrane vibration in the CDR is transmitted to the auditory nerve. Since the transduction of information that should be carried to the auditory cortex does not occur in the CDR, individuals with CDR(s) often complain that they have difficulty understanding speech and receive limited hearing aid benefit^4–9^. Despite the location of CDR(s) playing such an important role in fitting, the presence of CDR(s) cannot be identified using standard audiometric testing^10^.

Many investigators have used psychophysical tuning curves (PTCs) to identify CDRs^2,11–13^. A psychophysical tuning curve is a measure of how intense a masking stimulus needs to be in order to affect the ability of the listener to perceive a target signal (the probe). The tip of the PTC is defined as the frequency where the level of the masker required to affect detection of the probe tone is lowest. For subjects without CDR(s), the tip of the PTC (i.e., the frequency at which the masker level is lowest) should fall close to the signal (or probe) frequency. For subjects with CDR(s), the tips are shifted well away from the probe frequency.

While PTCs are considered the gold standard for detecting CDR(s), they are time-consuming to obtain and are rarely (if ever) measured in clinical settings. The threshold-equalizing noise (TEN) test was introduced in 2000 by Brian C. J. Moore for this purpose^1,3,14^. TEN is noise that has been shaped so that the masked threshold of a pure tone is the same for all frequencies from 250 to 10,000 Hz in NH subjects. When a tone is presented in a CDR, the masked threshold will be elevated because the TEN noise interferes with off-frequency listening. That means inner hair cells and neurons with characteristic frequencies different from that of the signal frequency are used for detection. Thus, CDRs are identified by masked thresholds that are much higher (e.g. >10 dB higher) than both the absolute threshold for the tone and the levels required to obtain a masked threshold from an individual without a CDR(s)^2^.

The TEN test is relatively simple and fast to administer and studies have shown that it can be an effective method for identifying CDRs in hearing-impaired listeners^1,3,14^. However, it requires behavioral responses from the subject which is inappropriate for infants/young children, individuals who are not inclined to cooperate or individuals with developmental delays or other cognitive challenges. In this study, we propose a non-behavioral, electrophysiologic method for identification of a CDR.

The acoustic change complex (ACC) is an auditory evoked potential that can be measured from adults or children in a passive listening paradigm^15–19^. It is a variant of the classic P1-N1-P2 onset response and it is elicited when the listener is able to detect a change in some aspect of an ongoing sound^16,20,21^. The ACC can also be evoked using speech stimuli^22^. ACC responses have been recorded successfully from normal–hearing listeners^16,23,24^, hearing–impaired listeners^25^, cochlear implant users^26,27^, and patients with auditory neuropathy spectrum disorder^28,29^. ACC responses have good test-retest reliability^30^. In this study we describe a method for using the ACC to identify CDR(s). We have adapted the TEN test for this purpose and refer to the stimuli we use for evoked potential testing as “modified TEN stimuli” and the objective version of the TEN test that we have developed “TEN-ACC”.

This report describes results from two different studies. The goal of the first study is to optimize the stimulation and recording procedures and results on the TEN-ACC test obtained from normal hearing listeners are described. The goal of the second study was to evaluate the feasibility of using the TEN-ACC test to detect CDR(s) in HI listeners.

## Methods

The two studies were conducted sequentially and approved by the Samsung Medical Center-Institutional Review Board. All participants signed an informed consent. This study was carried out in accordance with approved guidelines.

### Subjects

Thirty eight NH listeners, aged between 21 and 35 years (NH group, mean age = 26.2 years, SD = 3.43 years) of which 20 were male, participated in study 1. In study 2, 10 NH listeners (mean age = 24.1 years, SD = 1.97 years), 12 HI listeners without CDR (HI group, mean age = 52.42 years, SD = 11.30 years), and 4 HI listeners with CDR (CDR group, mean age = 46.00 years, SD = 9.42 years), a total of 26 NH and HI listeners participated of which 11 were male.

Pure-tone audiograms were obtained from all study participants. NH group was required to have audiometric thresholds no worse than 25 dB HL for octave frequencies from 250 to 8000 Hz. HI and CDR groups were required to have audiometric thresholds worse than 40 dB HL for octave frequencies 250 and 8000 Hz. The test ear for subjects in the NH and HI groups was selected randomly. The test ear for subjects in the CDR group was selected based on the results of the behavioral TEN test.

### Behavioral TEN test

The TEN test was developed and validated by Moore^14^. It measures the level required for a listener to detect the presence of a pure tone presented in TEN. Study participants completed the behavioral version of the TEN test in a soundproof booth using an audiometer (Orbiter 922, Madsen Electronics, Minnetonka, MN) and ER-3A insert earphones. TEN level was fixed at 60 dB HL/ERB_N_ for the NH group and 10 dB above the absolute threshold of each pure tone frequency for the HI and CDR groups. An adaptive procedure was used to measure masked pure tone thresholds. Pure tones between 500 and 4000 Hz were presented in the presence of noise. The level of the pure tone was decreased by 4 dB when participants had a correct response and increased by 2 dB when participants had an incorrect response until the masked threshold was identified.

### Electrophysiological TEN-ACC test

#### Modified TEN stimuli

**Figure 1:**
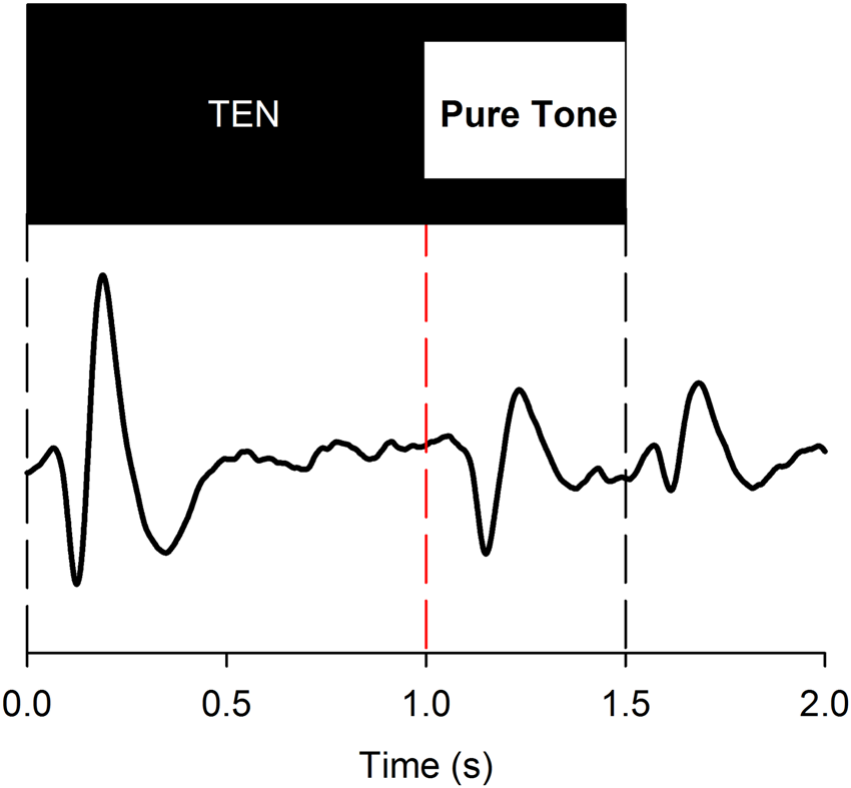
An examples of the modified threshold equalizing noise (TEN) stimulus in the second study. The upper panel is a block diagram of the stimulus pattern. Stimulation was made up of part TEN only and part pure tone embedded in TEN. The modified stimulus elicits onset (first dashed line), ACC (second red dashed line), and offset (last dashed line) responses. When the stimulus was presented and pure tones were added to TEN, rise/fall time of 5- or 10- ms was applied to the stimulus to avoid abrupt onset/offset artifacts.

Figure 1 shows the schematic of the stimuli used to evoke the ACC and a response from a NH group from the second study. An onset response is measured for the TEN presented for 1 second before the 1000 Hz pure tone is added. If the listener perceives the added pure tone, a second P1-N1-P2 response (the ACC) is recorded. A smaller offset response is also often measured.

The purpose of study 1 was to optimize stimulation parameters. As described in Table 1, two separate stimuli were evaluated. In study 1A, the duration of the modified TEN stimuli was 1 second. The first half of the stimulus consisted only the TEN. Pure tones of various frequencies and levels were added to the second half of the 1 second stimulus. A rise/fall time of 10 ms was applied to the stimulus in order to avoid abrupt onset/offset artifacts (spectral splatter). The inter-stimulus interval was one second, and the level of the TEN was fixed at 60 dB HL. The waveform recorded in study 1A represent the average of 150 stimulus presentations. In study 1B, the stimulus duration was increased to 2 seconds, but the number of sweeps was decreased from 150 to 120.

**Table 1:** The stimulation parameters used in 2 studies.

Participants	Study 1A	Study 1B	Study 2		
	NH		NH	HI without CDR	HI with CDR
TEN level	60 dB HL		60 dB HL	Audiometric θ + 10 dB	
Pure tone level (1 and 4 kHz): 3 dB step	−3~15 dB SNR	0~15 dB SNR	0~15 dB SNR	0~18 dB SNR	6~21 dB SNR
Total stimulus time	1 s	2 s	1.5 s		
Time for pure-tone stimulus	0.5 s	1 s	0.5 s		
Inter-stimulus interval	1 s	2 s	1.5 s		
Number of Sweeps	150	120	120		
Rise/fall time	10 ms		5 ms		

In study 2, the duration of the second portion of the stimulus that contained the pure tone was decreased from 1 second to 0.5 seconds resulting in a total stimulus duration of 1.5 seconds. The inter-stimulus interval was also decreased to 1.5 seconds, and the rise/fall time was decreased from 10 to 5 ms. The noise stimuli used for this modified TEN test were identical to those used for the behavioral test.

The level of the embedded pure-tones varied from −3 to 21 dB SNR in 3-dB steps for studies 1A, 1B and study 2. Stimulus conditions across the studies are summarized in Table 1.

In study 2, the stimulus level for the pure tones ranged from 0 to 15 dB SNR in NH group. In HI and CDR groups the presentation level for the pure tones ranged from 0 to 18 dB SNR and from 6 to 18 dB SNR, respectively. If needed, the tone level of 21 dB SNR is also conducted. Two pure tone frequencies (1 and 4 kHz) were used for the TEN-ACC test. The modified TEN stimuli were presented through the ER-3A insert earphones connected with a Neuroscan STIM system (NeuroScan, Inc., Herndon, VA). NH and HI group test ear was selected randomly and test ear of CDR group was selected accordingly based on the behavioral TEN test result.

#### Electrophysiological recordings

During the electrophysiological recording session, subjects were seated in a reclining chair in a soundproof booth and asked to relax and watch a captioned video to help them stay awake. The test environment was observed using an infrared camera placed inside the soundproof booth. If the subject moved or dozed off during recording, that specific condition was repeated after reinstructing the subject to not move or fall asleep.

Surface recording electrodes were placed at Cz, in accordance with the international 10–20 system^31^. Reference and ground electrodes were placed at the contralateral mastoid and forehead, respectively^25,26^. According to a previous study, this electrode montage results in the largest ACC amplitude recordings^16^. Eye movements were monitored using recording electrodes placed above and below the ipsilateral eye and lateral to both eyes. Electrode impedances were maintained below 5,000 ohms and inter-electrode impedance differences were kept below 2,000 ohms. Each ACC recording represented the average of 120–150 stimulus presentations.

EEG activity was amplified (x10) and band-pass filtered between 0.1 and 100 Hz before being sampled at a rate of 1,000 Hz. Once the data were collected, sweeps containing electro-ocular artifacts were excluded from the averaged recordings. EEG epochs were created using a 100 ms pre-stimulus baseline and a 1,500 ms, 2,500 ms or 2,900 ms post-stimulus interval for each condition. EEG epochs in which voltage exceed 100 μV were rejected from further procedure. After artifact rejection, EEG epochs were baseline corrected (−100 ms to 0 ms) and band-pass filtering from 1 Hz to 30 Hz (12 dB/octave) was applied. The filtered data were averaged for each stimulus level and for each subject. The smoothing of averaged waveform was conducted using a 40 ms boxcar filter. Finally, the data were averaged across subjects to create grand-mean average waveforms.

Two experienced audiologists individually analyzed the waveforms and determined the ACC responses and masked thresholds. Just one of two audiologists was blinded to the subject group, hence the presence of ACC response was decided when two audiologists reach identical judgment. In study 2, a root mean squared (RMS) amplitude ratio of ACC to noise floor was added to determine the ACC response. The windows for the ACC response and noise floor were from 1050 to 1250 ms and from 2800 to 2900 ms after onset, respectively. When the RMS amplitude of the ACC was higher than that of noise floor at least 50%, the ACC response was determined to be present. In summary, in study 1, only decision of audiologists was for determining presence of the ACC response. In study 2, the presence of the ACC response was determined based on both criteria mentioned above.

## Results

### Study 1

**Figure 2:**
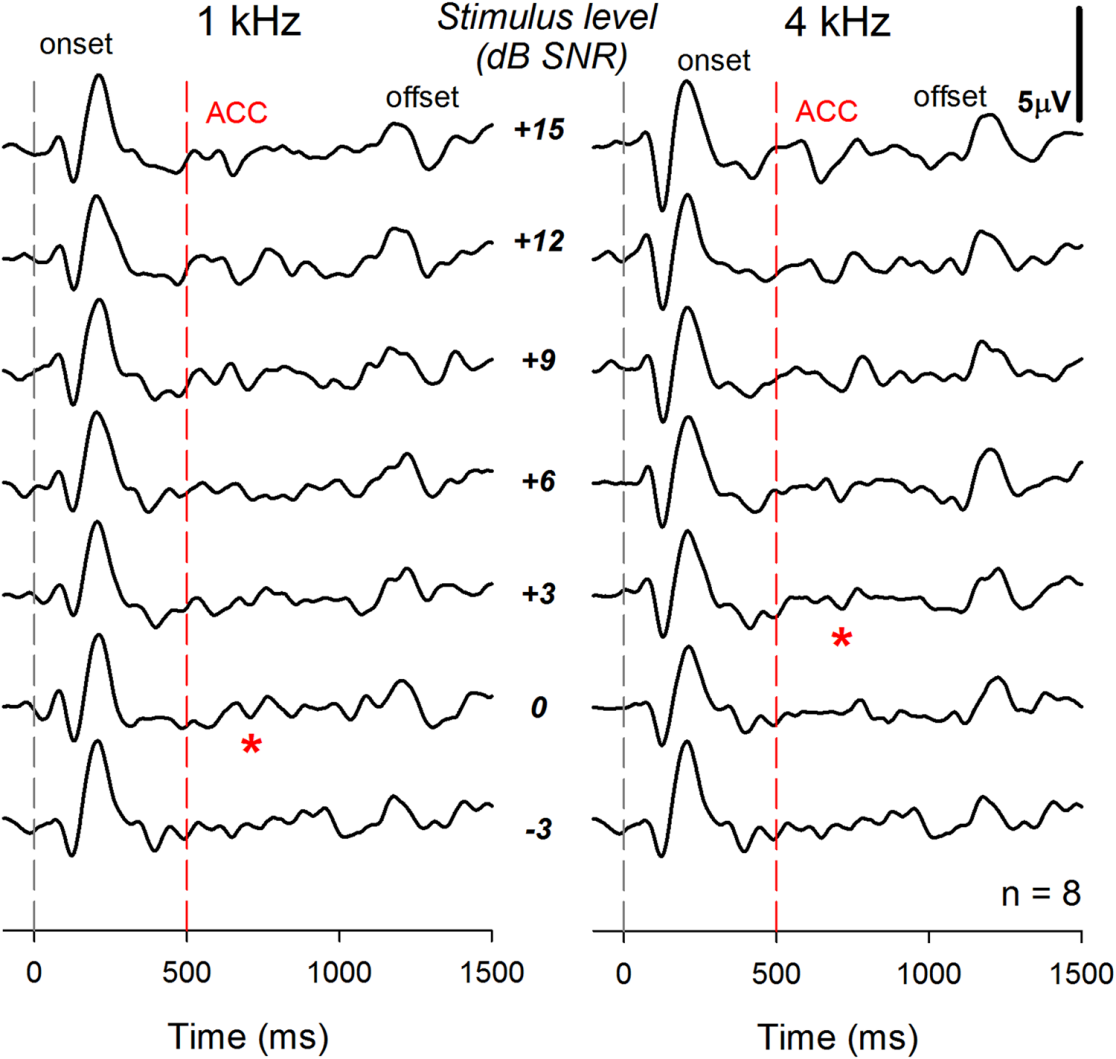
Grand average waveforms of NH group in study 1A were displayed. The gray dash line indicates onset time and the red dash line indicates time to present pure tones embedded in TEN. In study 1A, the number of subjects is eight. The asterisks indicate the masked threshold at 1 kHz and 4 kHz. Both electrophysiologically obtained thresholds were measured at 0 dB SNR and 3 dB SNR, respectively.

Figure 2 displays grand average waveforms obtained for each SNR from NH group who participated in study 1A (n = 8). Onset responses are shown. After half of one second, a 1000 Hz tone is added to the noise. ACC responses are recorded when the SNR is favorable (e.g. >3 dB). Amplitude of the ACC increases as the SNR is increased from −3 dB to +15 dB. The same pattern is observed for the 4 kHz tones. The TEN is turned off at 1000 ms and an offset response is also apparent in the grand mean waveforms. Note that masked thresholds for the TEN-ACC test at 1 kHz and 4 kHz were +0 dB and +3 dB SNR, respectively. This is lower than the +10 dB SNR criterion used to identify a CDR in the behavioral TEN test. While ACC responses are apparent in the grand mean averages, it was difficult to identify the ACC in the individual waveforms.

**Figure 3:**
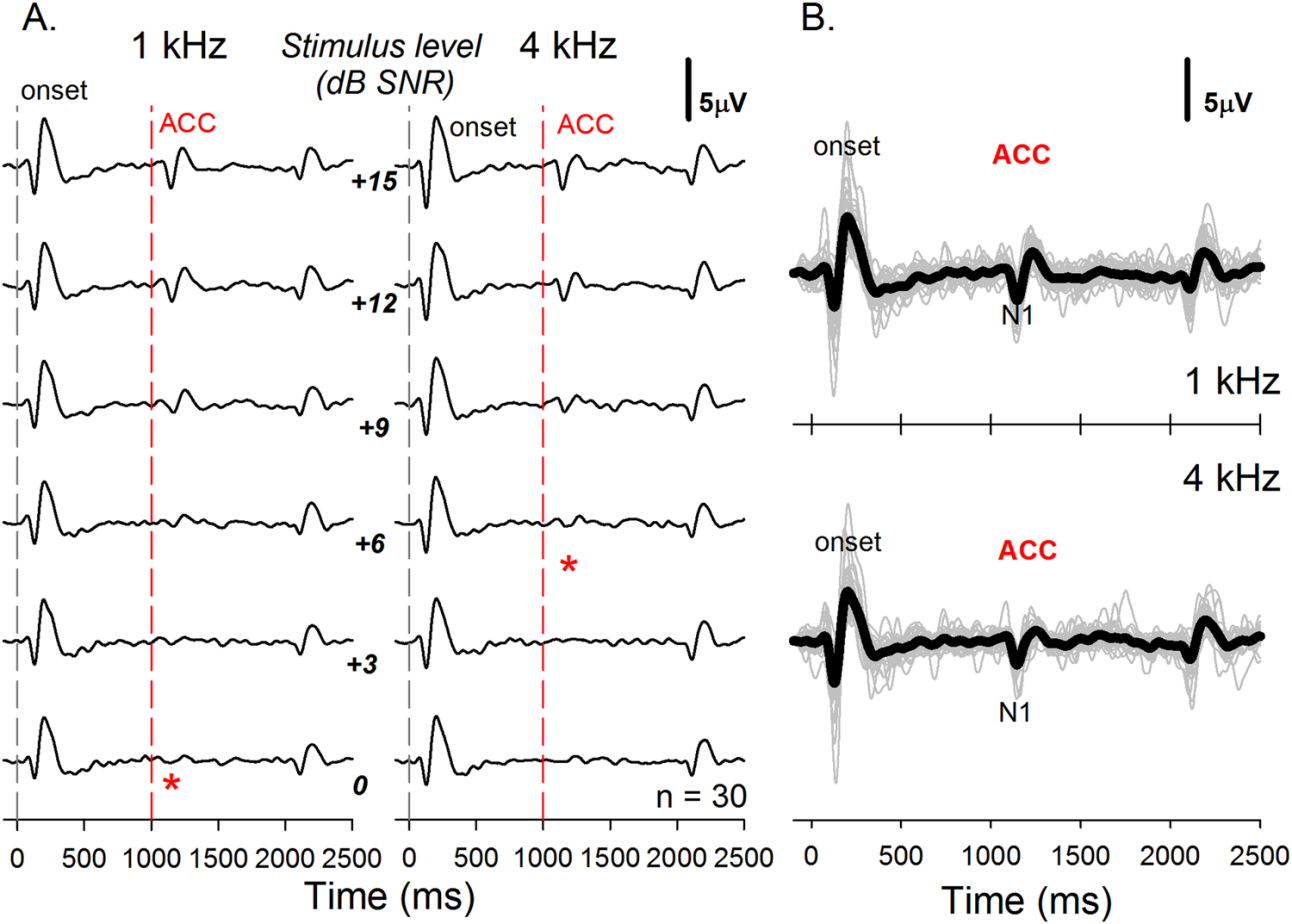
The graph shows grand-average waveform of normal-hearing subjects in study 1B. The number of subject in study 1B is thirty. (**A**) The asterisks indicate the masked threshold of each pure tone stimulus. The masked thresholds were 0 dB SNR and 6 dB SNR at 1 kHz and 4 kHz, respectively. (**B**) The waveform shows ACC responses with maximum stimulus level (15 dB SNR) at 1 kHz and 4 kHz. The ACC responses were clearly evoked in all normal-hearing subjects.

Results of study 1B are displayed in Fig. 3A,B. This study involved using longer stimulation and inter-stimulus intervals and 120 sweeps (see Table 1). These choices helped minimize the effects of adaptation in the data. ACC responses are recorded at SNRs as low as 0 dB for the 1 kHz tone and 6 dB for the 4 kHz tone. Figure 3B shows individual ACC waveforms superimposed on the grand mean averages. Averages of behaviorally obtained masked thresholds (TEN thresholds) in study 1B were −0.20 (±1.61) dB SNR and −0.93 (±1.46) dB SNR at 1 kHz and 4 kHz, respectively. Averages of masked thresholds obtained using the ACC response (TEN-ACC threshold) were 6.10 (±3.57) dB SNR and 8.10 (±3.36) dB SNR at 1 kHz and 4 kHz, respectively. While the electrophysiological measures are not as sensitive as the behavioral measures, the TEN-ACC thresholds were below the 10 dB criterion of CDR(s) proposed by Moore *et al*.^32^.

### Study 2

**Figure 4:**
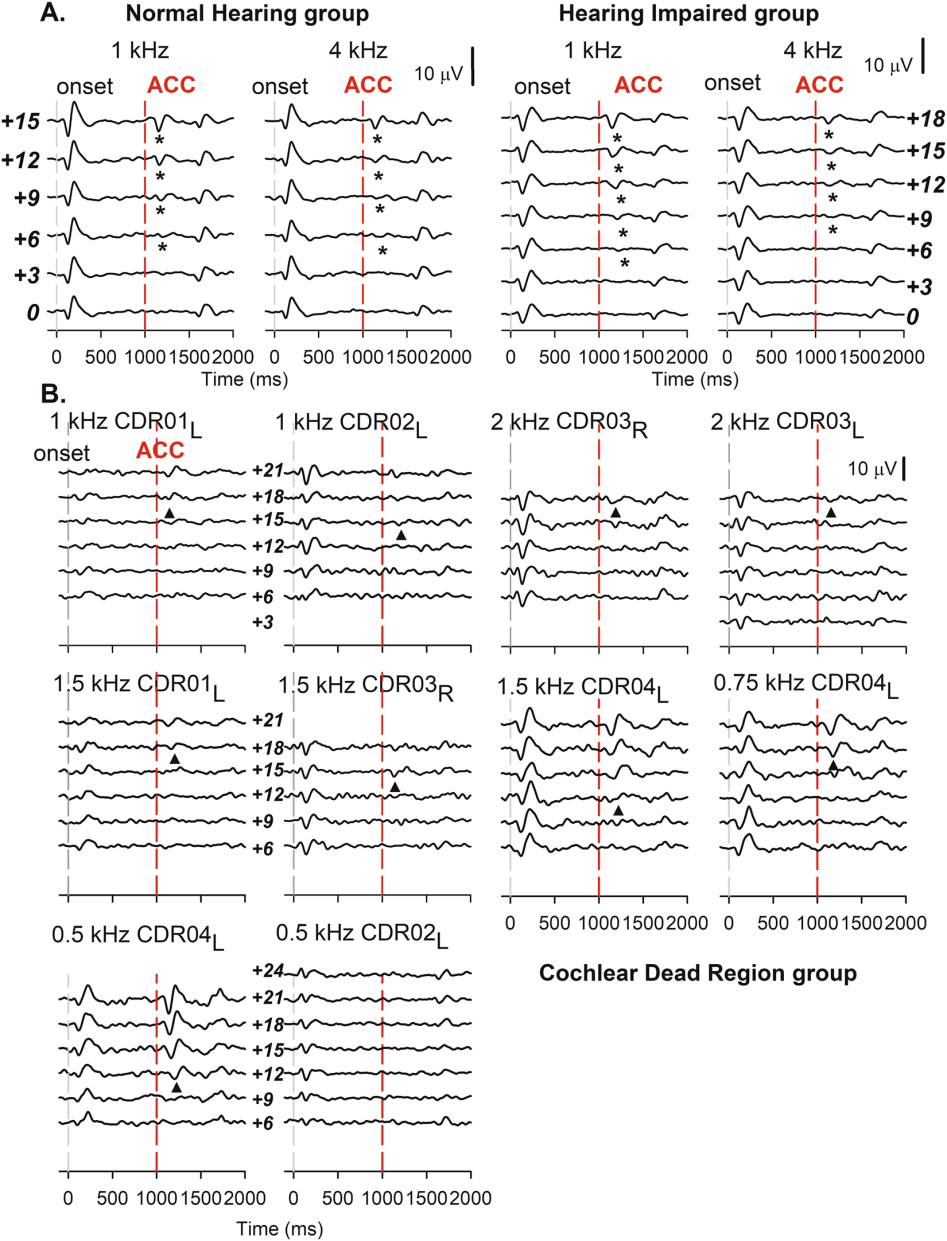
Grand-average waveforms of NH and HI groups in study 2 are displayed. The gray dash line indicates onset time and the red dash line indicates time to present pure tones embedded in TEN. (**A**) The asterisks indicate the N1 peaks of the grand-average ACC responses of normal-hearing and HI participants without CDR. (**B**) The thresholds of ACC responses of each participant with CDR were indicated by a triangle symbol. The thresholds were observed to be above 9 dB SNR.

In study 2, EEG data were obtained from NH group as well as HI and CDR groups. The time window for Fig. 4 extends from −100 ms to 2000 ms to show responses clearly. Figure 4A shows grand average waveforms recorded from the NH group and the HI group who did not have evidence of a CDR. Grand mean averages for both the 1 kHz and the 4 kHz stimuli are shown at each SNR. The grand mean waveforms suggest that masked thresholds for the 1 kHz and 4 kHz were 6 dB SNR and 6 dB SNR, respectively, for the NH group and 6 dB SNR and 9 dB SNR, respectively, for the HI group.

Figure 4B shows a series of individual waveforms recorded from four individuals with CDR(s). TEN-ACC thresholds ranged from 12 to 24 dB SNR for these four subjects. These thresholds are considerably higher than thresholds for individuals included in the NH and HI groups.

Table 2 shows the mean behavioral (TEN) and electrophysiological (TEN-ACC) thresholds for all three subject groups. Behavioral thresholds are clearly lower than electrophysiologic thresholds. The differences between the mean masked thresholds measured behaviorally and using the ACC for the NH group were 6.6 and 8.4 dB SNR at 1 kHz (Wilcoxon signed rank test, z = 2.812, p = 0.002) and 4 kHz (t = −7.145, p < 0.001), respectively. The differences between the masked thresholds measured using behavioral and electrophysiological tests were 5.33 and 6.08 dB SNR at 1 kHz (t = −4.959, p < 0.001) and 4 kHz (t = −4.906, p < 0.001), respectively for subjects in the HI group. These differences were statistically significant. For the four subjects with CDR(s), TEN-ACC thresholds were recorded using stimulus frequencies in a CDR as determined based on behavioral testing (i.e. 0.5 kHz, 0.75 kHz, 1 kHz, 1.5 kHz, and 2 kHz). The electrophysiologic measures of TEN-ACC threshold for these four study participants were higher than results obtained using behavioral testing techniques. The difference between the masked thresholds across all stimulus frequencies measured using behavioral and electrophysiological tests was 5.2 dB SNR (t = −7.839, p < 0.001).

**Table 2:** Comparison of the threshold of behavioral TEN(T) test and electrophysiological TEN-ACC(TA) test for all subject groups.

		1 kHz					4 kHz				
		T_θ (SNR)		TA_θ (SNR)			T_θ (SNR)			TA_θ (SNR)	
NH (e = 10)	Average	0.00		6.60			−1.80			6.60	
	SD	2.49		3.69			1.14			4.20	
HI (e = 12)	Average	3.17		8.50			2.67			8.75	
	SD	2.89		2.81			1.97			4.14	
		**0.5 kHz (2)**		**0.75 kHz (1)**		**1 kHz (2)**		**1.5 kHz (3)**		**2 kHz (2)**	
		**T_θ**	**TA_θ**	**T_θ**	**TA_θ**	**T_θ**	**TA_θ**	**T_θ**	**TA_θ**	**T_θ**	**TA_θ**
CDR (e = 5)	Average	15	18	12	18	11	16.5	10	15	11	18

The ‘e’ represents the number of ears tested in this study. Number in parenthesis refers to number of subjects participated in each frequency condition of pure tone stimulus.

**Figure 5:**
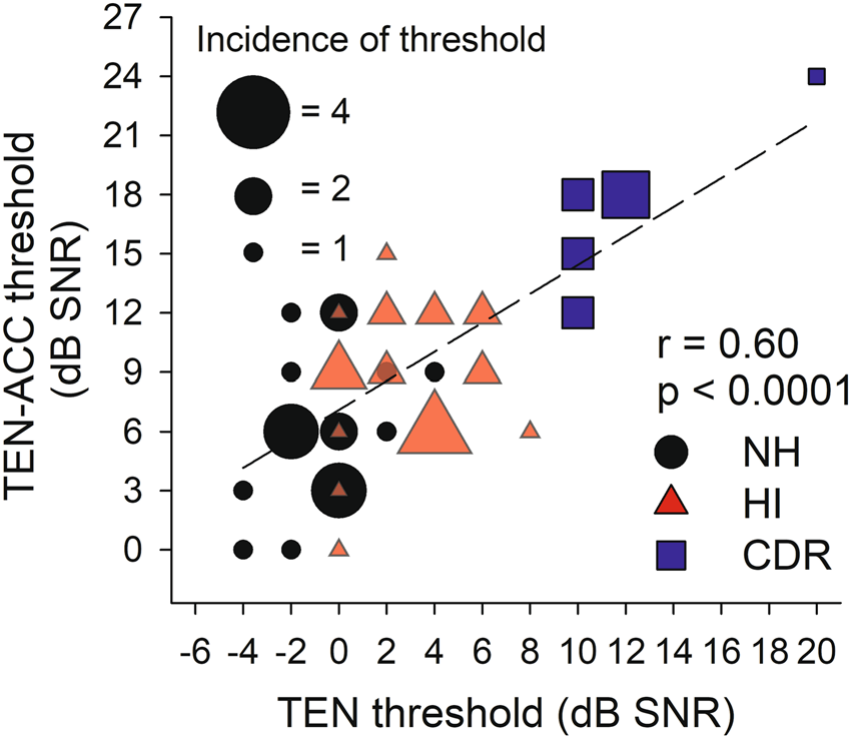
The graph shows correlation between behaviorally and electrophysiologically obtained thresholds. The two thresholds showed moderate-to-strong correlation (r = 0.60, p < 0.0001). The black symbols indicate data of normal subjects, the red symbols indicate results of hearing-impaired subjects without CDR, and the blue symbols indicate results of hearing-impaired subjects with CDR.

Figure 5 is a scatterplot showing the relationship between the results of the TEN test and the TEN-ACC test. Different symbols show results from the three subject groups. Linear regression analysis was used to examine the strength of the relationship between the evoked potential and behavioral measures. For this analysis, results of both TEN test and TEN-ACC test for all 3 groups at all stimulus frequencies are combined. The dashed line shows the results of the linear regression. The correlation between the two measures was found to be statistically significant (DF = 53, *r = *0.60, *p* < 0.0001).

**Figure 6:**
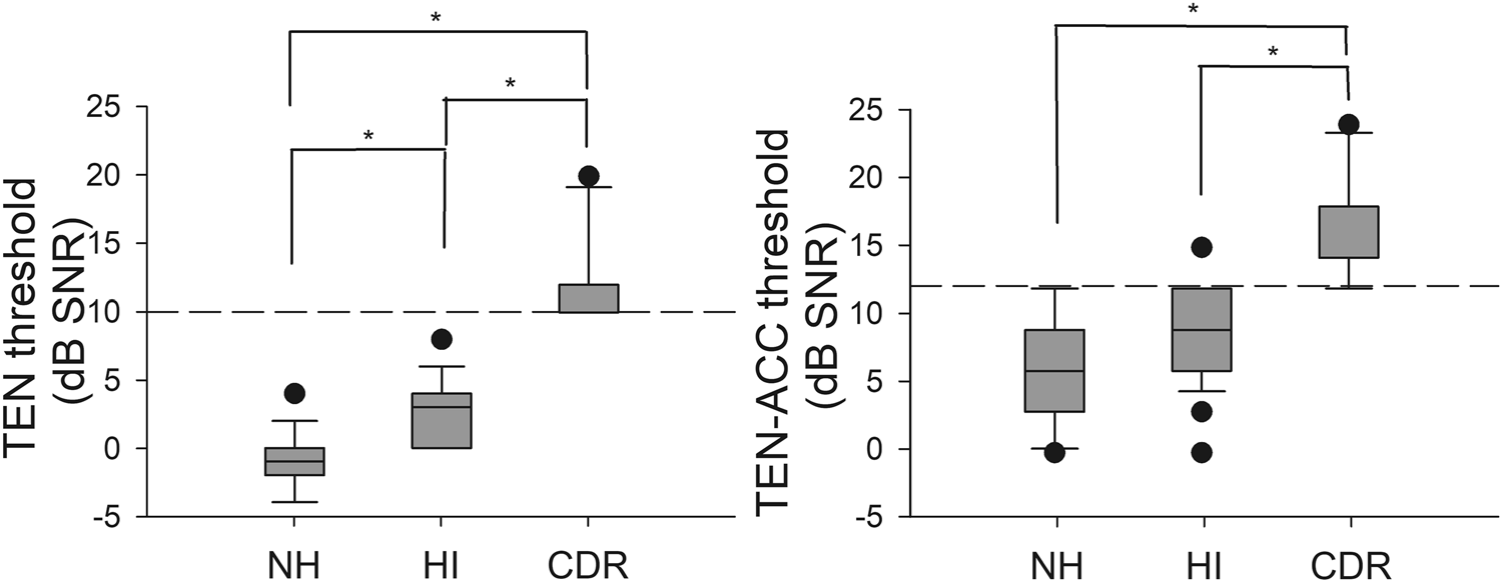
The box plots indicate the range of thresholds along the different subject groups and tests. The dashed line indicates the criterion (10 dB) and potential criterion (12 dB) to decide the presence of the cochlear dead region in behavioral TEN test and electrophysiological TEN-ACC test, respectively. The results of behavioral test are presented on the left panel. The right panel displays the threshold range from the electrophysiological test. In CDR group, the threshold range was observed to be higher than the 10 dB criterion in behavioral TEN test, while in electrophysiologic TEN-ACC test, the range of thresholds was higher than 12 dB SNR.

Figure 6 shows the same data as Fig. 5 arranged in a box plot. Results obtained using the TEN test and TEN-ACC test are shown on the left and right, respectively. The dashed line on the left panel indicates criterion to identify presence of CDR in a behavioral TEN test. The dashed line on the right panel indicates potential criterion that we suggest to confirm the presence of CDR in an electrophysiologic TEN-ACC test. The line in the box indicates median value and the dot indicates outlier of data. Only the thresholds of CDR group were higher than 10 dB, criterion for CDR(s), in behavioral TEN test and higher than 12 dB in electrophysiological TEN-ACC test. One way analysis of variance was used to identify statistically threshold difference for the groups. In behavioral TEN test (Kruskal-wallis one way ANOVA), difference of thresholds between the three groups were statistically significant (*p* < 0.001). Post-hoc analysis indicated the mean TEN threshold of CDR group was larger than those of NH (*p* < 0.05) and HI (*p* < 0.05) groups. In addition, the mean TEN threshold of HI group was greater than that of NH group (*p* < 0.05). Statistically significant group effects were obtained for the TEN-ACC test (*p* < 0.001). Post-hoc analysis revealed the mean TEN-ACC threshold of CDR group was greater than those of NH (*p* < 0.05) and HI (*p* < 0.05) groups. Furthermore, mean TEN-ACC threshold of HI group was larger than that of NH group, but the difference was not statistically significant.

## Discussion

Results of this study suggest that it is possible to identify CDRs using electrophysiological methods. The first study allowed us to optimize the parameters for the TEN-ACC test by analyzing the effect different stimulation parameters had on ACC responses. Results of the second study showed that ACC responses can be recorded from individuals with CDRs. Behavioral test results also showed threshold elevation.

The ACC has been used in several previous studies as an electrophysiologic measure of discrimination of changes in the spectral, temporal, and/or intensity of an acoustic signal^23,27,33^. In this study, we extend that work to demonstrate that the ACC could also be used as a proxy for behavioral testing to identify CDR(s).

In study 1, the robust ACC response was well elicited by pure tones embedded in TEN in all subjects with NH. Additionally, the ACC response was affected by pure tone stimulus level, suggesting that it is feasible to decide the threshold for pure tone detection in background TEN using an electrophysiological approach. We optimized the test condition by making sure the newly developed TEN-ACC test is time-efficient and reliable to be widely applicable in clinical settings. Thus, various stimulus durations, stimulus levels, inter-stimulus intervals, rise/fall times, and sweep numbers were applied in study 1 and 2.

In study 1A, we obtained a promising result that the modified TEN stimuli used in study 1A could elicit ACC responses. Clear ACC responses can be seen in the grand-average waveform, but individual ACC responses were very hard to detect due to noise. We speculate that this is due to the onset response from TEN overriding the ACC response as a result of short stimulus duration. Therefore, in study 1B, the stimulation duration of the TEN-only and pure tone added TEN portions are increased to 1 second from 0.5 seconds for each stimulus in order to avoid the influence TEN onset response has on the ACC response. Since increasing of stimulus duration is time-consuming, the number of sweeps was decreased to reduce total test time. As a result, the ACC responses were observed not only in the grand average waveform, but also in individual waveforms in all participants.

In addition, in study 2, a raised linear ramp of 5-ms rise and fall was used to evoke the ACC as previous studies revealed that spectral splatter can be successfully reduced with a 5-ms rise and fall time^34^. Furthermore, the duration of a pure tone added TEN was decreased to 0.5 seconds for efficiency while the TEN only part was kept at 1 second to not affect the ACC.

In study 2, the ACC response was obtained for all three groups: NH, HI, and CDR. The ACC response was also observed in both HI and CDR groups. Statistical analysis on the obtained TEN-ACC test data with one-way analysis of variance clearly showed differences between the three groups. Particularly, the TEN-ACC threshold in the CDR group was significantly higher than those in NH and HI groups, and the absolute value of TEN-ACC threshold in the CDR group was around or above 12 dB SNR.

For NH and HI listeners without CDR(s), the masked threshold of the TEN test is similar or somewhat higher than that of TEN level. If a HI listener has CDR, the masked threshold is 10 dB or greater than TEN level and audiometric threshold. (In this study, we set the TEN level 10 dB higher than the audiometric threshold so that the second criterion of CDR was automatically satisfied). As the masked threshold is 10 dB or greater than the TEN level in the behavioral TEN test, a diagnostic criterion for CDR, our results suggest that CDR can be detected using the electrophysiological approach.

With the TEN-ACC test, the absolute values of masked thresholds were somewhat higher than those obtained from the behavioral TEN test, but have a similar trend for all groups. Although thresholds of the two different measurements were not quite similar, a moderate-to-strong correlation between the TEN threshold and TEN-ACC threshold exists and is shown in Fig. 5. This indicates that the TEN-ACC test is quite reliable in obtaining masked thresholds.

**Figure 7:**
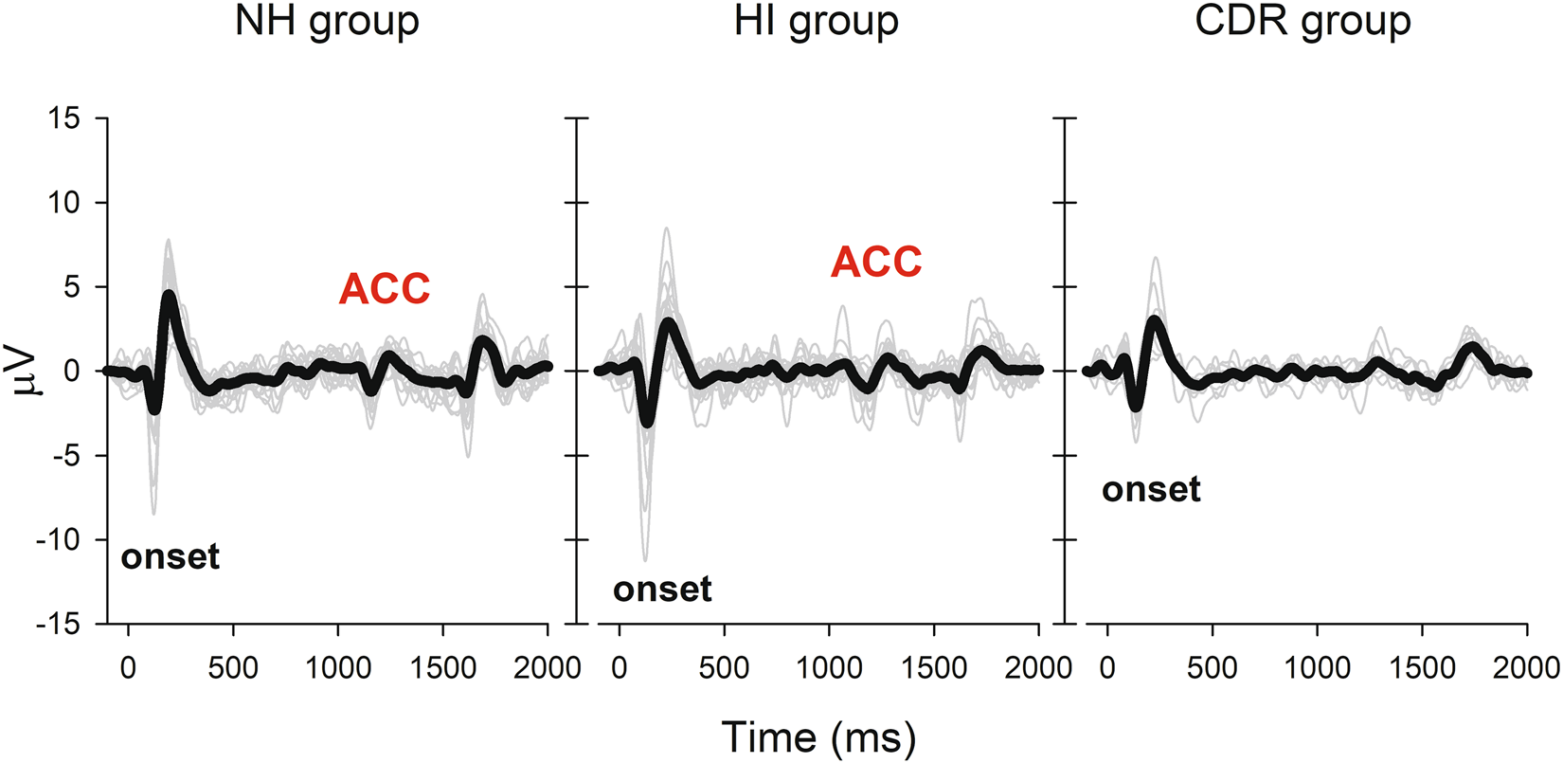
The graphs show the individual and grand mean waveform graphs for all three groups at 12 dB SNR stimulus level. The left panel displays the waveform of NH group, the middle panel shows the waveforms of HI group, and the right panel shows the CDR group waveforms. The gray lines are for the individual waveforms, and thick black lines indicate the grand mean waveforms.

Figure 7 shows the individual (gray line) and grand mean waveform (black line) graphs for all 3 groups at 12 dB SNR stimulus level. In the NH and HI groups, there were ACC responses in all participants except one participant. In the CDR group, only two out of ten results show the ACC response. In other words, the objective TEN-ACC test to determine ACC response has the specificity of 98% and the sensitivity of 80%.

Although the subject population that was tested in this study was comprised of cooperative adults, objective measures, in general, are more applicable for difficult-to-test or pediatric populations. The CDR occurrence is relatively common in adolescents with a longstanding severe to profound hearing loss^35^. Almost 70% of adolescents met the criteria for CDR in at least one ear. Generally, the morphology of auditory evoked potential has a maturation process with age or hearing impairment. The ACC, a type of evoked potentials, could also have the morphology of a delay in latency depending on age or presence of hearing loss. Martinez (2013) showed that the ACC response to vowel change was evident in children with or without hearing impairments ranging from 2 years and 3 months to 6 years and 3 months^36^. Most children clearly had a P1-N2 response to the changes of vowels. Therefore, we expect the ACC response to the modified TEN stimuli used in this study could occur in young children with hearing loss. Additionally, further studies applying the TEN-ACC test to uncooperative children or infants would be necessary to identify sensitivity and whether the result agrees with the behavioral test.

Previous studies also tried to objectively measure PTCs using auditory brainstem response (PTC-ABR) or auditory steady state response (PTC-ASSR). However, this was quite time-consuming, requiring at least 2 hours per tuning curve^37^. In this study, the time required to complete the TEN-ACC test per frequency was approximately 40 minutes. This can be further reduced by decreasing the number of SNRs that were tested. For example, rather than a full TEN-ACC test measured at multiple SNRs, it can be measured at a single SNR (e.g. +12 dB SNR) as a way of screening. A single SNR condition of TEN-ACC test in study 2 requires just 6 minutes. If a clear ACC is recorded, it would indicate an absence of CDR. However, the lack of response would indicate an existence of CDR at that test frequency. Therefore, the objective approach of TEN-ACC seems to be more feasible in a clinical setting in terms of time-efficiency. In this study, the number of sweeps was 120 to reduce testing time. Although this number is enough to observe ACC responses at the supra-threshold level, for screening purpose, 120 repetitions may not be sufficient enough to clearly determine the presence of ACC responses. Therefore, it is suggested to increase the number of sweeps more than two times.

As far as we know, this is the first study to attempt to objectively detect CDR using the ACC. The results obtained in this study demonstrated the potential of TEN-ACC as an objective approach for detection of CDR in a clinical setting. However, further studies with the larger population, using finer step sizes of SNRs, and testing more frequencies are needed to confirm the feasibility of using this test in clinical practice.
